# Prescribing patterns of SGLT-2 inhibitors for patients with heart failure: A two-center analysis

**DOI:** 10.1016/j.ahjo.2023.100286

**Published:** 2023-03-07

**Authors:** Teja Chakrala, Roshni O. Prakash, Justin Kim, Hanzhi Gao, Umar Ghaffar, Jaymin Patel, Alex Parker, Bhagwan Dass

**Affiliations:** a Department of Medicine, University of Florida, Gainesville, FL, United States of America; b Department of Biostatistics, University of Florida, Gainesville, FL, United States of America; c Division of Hospital Medicine, University of Florida, Gainesville, FL, United States of America; d Division of Cardiovascular Medicine, University of Florida, Gainesville, FL, United States of America

**Keywords:** Prescribing patterns, Sodium-glucose cotransporter-2 inhibitors (SGLT-2i), Heart failure, Heart failure with preserved ejection fraction

## Abstract

**Background:**

Sodium glucose co-transporter 2 inhibitors (SGLT2i) have been proven to reduce the combined risk of cardiovascular death and hospitalizations in patients with heart failure (HF), irrespective of the presence or absence of diabetes. Despite class 1 and class 2A recommendations for their usage in HF with reduced ejection fraction (HFrEF) and HF with preserved ejection fraction (HFpEF) respectively by the American College of Cardiology, their prescription rate has remained low.

**Objective:**

The aim of this study is to analyze SGLT2i prescription patterns at two academic institutions, with the goal of identifying barriers to implementation.

**Design:**

A two-center retrospective analysis was conducted on patients ≥18 years old with a diagnosis of heart failure who were admitted to one of two hospital systems between 5/1/21 and 5/31/22. Patients with an eGFR ≥20 mL/min/1.73m^2^ and BNP ≥ 100 pg/mL were included.

**Results:**

SGLT2i was prescribed in only 19 out of 1081 HFpEF patients (1.8 %) and 51 out of 1596 HFrEF patients (3.2 %). A majority of SGLT2i prescriptions for the HFpEF population came from general medicine services (57.9 %) after obtaining approval from a cardiologist, which was required at our institutions. Adverse effects such as hypoglycemia and urinary tract infections were not significantly associated with SGLT2i use.

**Conclusions:**

Despite proven benefits of this class of medications as witnessed in large-scale clinical trials, the implementation of this drug class continues to be low.

## 1 Introduction

Heart failure is a chronic, progressive condition, characterized by episodic worsening of symptoms that results in frequent hospitalizations and significant healthcare costs [1], [2], [3]. Current HF guidelines recommend aggressive risk factor control in addition to initiation of several different classes of medications depending on the specific HF subtype. In patients with HFrEF, current clinical guidelines recommend patients be treated with quadruple therapy, which is a combination of medications consisting of a beta blocker, an angiotensin receptor-neprilysin inhibitor (ARNI), a mineralocorticoid/aldosterone receptor antagonist (MRA), along with an SGLT2i [4]. However, treatment options for patients with HFpEF have been limited.

The SGLT2i class of medication has been shown to be beneficial in the reduction of HF hospitalizations in patients with type 2 diabetes (T2DM) and chronic kidney disease (CKD). Several large-scale trials have demonstrated this benefit to be applicable to all heart failure populations. The benefit of SGLT2i in HF patients is thought to occur due to several mechanisms. First, they promote osmotic diuresis and natriuresis in patients with and without diabetes, reducing preload by plasma volume contraction [5]. Another hypothesis is that these medications may inhibit the sodium‑hydrogen exchanger 1 isoform in the myocardium, reducing cytoplasmic sodium and calcium concentrations [6]. The DAPA-HF clinical trial showed that the addition of dapagliflozin resulted in a reduced risk of death from cardiovascular causes in patients with HFrEF and was subsequently recommended as an addition to guideline-directed medical therapy (GDMT) in 2019. The EMPEROR-Reduced (2020) and EMPEROR-Preserved (2021) trials were multi-center landmark studies that investigated the effects of empagliflozin, another SGLT2i, in patients with HFrEF and HFpEF respectively [7], [8]. In both trials, the demonstrated value of SGLT2i was a decrease in the combined risk of cardiovascular death and hospitalizations for heart failure, regardless of the presence or absence of diabetes [7], [8]. However, since their publication, only a small fraction of patients in the United States have been prescribed the SGLT2i class of medication for treatment of heart failure [1], [4], [9].

SGLT2i appear to reduce overall mortality in people with diabetes and established cardiovascular disease (CVD). Several additional studies have confirmed the efficacy of SGLT2i in patients with heart failure [10]. This drug class has been associated with a 14 % relative reduction in the risk of all-cause mortality and cardiovascular mortality and has also reduced the risk of heart failure hospitalizations by 31 % [11]. In a meta-analysis of five trials comparing an SGLT2i with placebo in individuals with diabetes and CVD, SGLT2i reduced the risk of all-cause mortality (96 versus 113 per 1000 persons; OR 0.84, 95 % CI 0.74–0.96) [12].

Despite the growing evidence in favor of the utilization of SGLT2i in HF therapy, there is an institutional delay in its implementation. Our study investigates the multitude of factors that contribute to the use of SGLT2i in patients with HFpEF or HFrEF with and without diabetes, within two large academic centers.

## 2 Methods

A two-center study was performed with approval from the University of Florida Quality Improvement Project Registry (QIPR). It was conducted on patients admitted to the University of Florida Gainesville and Jacksonville hospital systems between May 1, 2021 and May 31, 2022. This period was chosen because the EMPEROR-Reduced and EMPEROR-Preserved Trials were published in October 2020 and October 2021 respectively, allowing time for the results of these trials to be employed, and May 2022 was the latest data available at the time of analysis.

We included patients ≥18 years of age with a diagnosis of HF that were admitted to the UF Health Shands and UF Health Jacksonville hospitals between 5/1/2021–5/31/2022, had a HF exacerbation hospitalization within the past 12 months, and a B-type natriuretic peptide (BNP) ≥ 100 pg/mL. Patients with a diagnosis of acute coronary syndrome, transient ischemic attack, orthotopic heart transplant (OHT) candidate or OHT recipient, and eGFR <20 mL/min/1.73 m2 were excluded. The Integrated Data Repository (IDR) team obtained a list of patients that met both our inclusion and exclusion criteria. Thereafter, the following variables were obtained from patient charts with assistance from IDR: medical record number, age, sex, body mass index, problem list, vitals during admission, hospital encounter, date of discharge for each encounter, 30-day readmission rate, admitting service, consulting services, medication list with start dates, laboratory values including complete blood count (CBC), basic and/or comprehensive metabolic panels (BMP, CMP), estimated glomerular filtration rate (eGFR), hemoglobin a1c% (HbA1c), BNP, urinalysis, ejection fraction on most recent transthoracic echocardiogram, and date of death (if applicable). In our study, HFrEF was defined as patients with an EF ≤ 45 % and HFpEF as those with an EF >45 % [13].

Demographics and clinical variables were compared between patients with HFrEF and HFpEF, and patients on an SGLT2i and not on an SGLT2i using two-sample *t*-tests, Wilcoxon-Mann-Whitney tests, Pearson's Chi-squared tests, or Fisher's exact tests as appropriate. The tests were at the two-tailed 0.05 significance level. Analyses were performed to examine the sensitivity of the main results along with the results comparison between the HFpEF and HFrEF subgroups.

## 3 Results

### 3.1 Demographics

A total of 3548 patient encounters for heart failure were identified for 2677 unique patients. Of the total number of encounters, 1426 were admissions for HFpEF and 2122 were admissions for HFrEF with 1081 unique patients having HFpEF and 1596 unique patients having HFrEF respectively. The average left ventricular ejection fraction (LVEF) in the HFrEF subgroup was 24.39 % versus 59.83 % in the HFpEF subgroup (p < 0.0001). BNP at baseline and eGFR were higher in patients with HFrEF when compared to HFpEF (1207.81 vs. 611.02, p < 0.0001, 54.60 vs. 52.89, p = 0.0082). There was no statistically significant difference between length of stay and baseline HbA1c of the two groups as depicted in Table 1. A higher proportion of the HFrEF population was admitted to a cardiology service at their baseline visit compared to the HFpEF group (565 [35.4 %] vs. 150 [13.9 %], p < 0.0001) and HFrEF patients had a higher 180-day readmission rate (318 [19.9 %] vs. 157 [14.5 %], p = 0.0004) when compared to HFpEF patients. The HFrEF group had a lower percentage of patients with T2DM (729 [45.7 %] vs. 540 (50.0 %), p = 0.0328), but a higher percentage of patients that were on SGLT2i when compared to those with HFpEF (51 [3.2 %] vs. 19 (1.8 %), p = 0.0305). There were no differences observed with differing doses of SGLT2i. In addition, there were more patients with HFrEF who were on a beta blocker, MRA, ACEi, and an ARNI (sacubitril-valsartan) when compared to patients with HFpEF as shown in Table 1. However, no significant difference was noted on the number of patients on an ARB.

**Table 1:** Characteristics of Patients with HFrEF and HFpEF.

Variable	Level	All	HFpEF	HFrEF	*P*-value
Number of patients		2677	1081	1596	
Average number of encounters		1.33 ± 0.89	1.25 ± 0.71	1.38 ± 0.99	<0.0001
Length of stay (days) at baseline visit		8.25 ± 9.18	8.07 ± 8.05	8.37 ± 9.87	0.3853
BNP at baseline visit		973.28 ± 1032.11	611.02 ± 672.37	1207.81 ± 1150.27	<0.0001
Hemoglobin A1c at baseline visit		6.62 ± 1.81	6.52 ± 1.64	6.68 ± 1.90	0.096
EGFR at baseline visit		53.91 ± 16.53	52.89 ± 16.31	54.60 ± 16.66	0.0082
LVEF at baseline visit		38.82 ± 20.26	59.83 ± 7.58	24.39 ± 11.88	<0.0001
Admit Svc Division at baseline visit	General Medicine	1678 (62.7 %)	828 (76.6 %)	850 (53.3 %)	<0.0001
	Cardiology	715 (26.7 %)	150 (13.9 %)	565 (35.4 %)	
	Family Medicine	80 (3.0 %)	35 (3.2 %)	45 (2.8 %)	
	Other	204 (7.6 %)	68 (6.3 %)	136 (8.5 %)	
180-Day Readmission	N	2202 (82.3 %)	924 (85.5 %)	1278 (80.1 %)	0.0004
	Y	475 (17.7 %)	157 (14.5 %)	318 (19.9 %)	
SSDI Death	N	2641 (98.7 %)	1072 (99.2 %)	1569 (98.3 %)	0.085
	Y	36 (1.3 %)	9 (0.8 %)	27 (1.7 %)	
LVEF at baseline visit		1110 (41.5 %)	452 (41.8 %)	658 (41.2 %)	<0.0001
Diabetes	N	1408 (52.6 %)	541 (50.0 %)	867 (54.3 %)	0.0328
	Y	1269 (47.4 %)	540 (50.0 %)	729 (45.7 %)	
SGLT2 Inhibitors at any visit	N	2607 (97.4 %)	1062 (98.2 %)	1545 (96.8 %)	0.0305
	Y	70 (2.6 %)	19 (1.8 %)	51 (3.2 %)	
Empagliflozin at any visit	N	2 (2.9 %)	0 (0 %)	2 (3.9 %)	0.0463
	Y	68 (97.1 %)	19 (100.00 %)	49 (96.1 %)	
Dapagliflozin at any visit	N	67 (95.7 %)	19 (100.0 %)	48 (94.1 %)	0.2775
	Y	3 (4.3 %)	0 (0 %)	3 (5.9 %)	
Empagliflozin Dose (mg)	10	65 (95.6 %)	19 (100.0 %)	46 (93.9 %)	0.5542
	25	3 (4.4 %)	0 (0 %)	3 (6.1 %)	
Alternative Medications (MRA, ACE_I, ARB, and Entresto)	N	308 (11.5 %)	176 (16.3 %)	132 (8.3 %)	<0.0001
	Y	2369 (88.5 %)	905 (83.7 %)	1464 (91.7 %)	
Beta blocker	N	532 (19.9 %)	319 (29.5 %)	213 (13.3 %)	<0.0001
	Y	2145 (80.1 %)	762 (70.5 %)	1383 (86.7 %)	
MRA	N	2173 (81.2 %)	925 (85.6 %)	1248 (78.2 %)	<0.0001
	Y	504 (18.8 %)	156 (14.4 %)	348 (21.8 %)	
ACE_I	N	1691 (63.2 %)	782 (72.3 %)	909 (57.0 %)	<0.0001
	Y	986 (36.8 %)	299 (27.7 %)	687 (43.0 %)	
ARB	N	2181 (81.5 %)	877 (81.1 %)	1304 (81.7 %)	0.7448
	Y	496 (18.5 %)	204 (18.9 %)	292 (18.3 %)	
Sacubitril-Valsartan or Entresto	N	2453 (91.6 %)	1064 (98.4 %)	1389 (87.0 %)	<0.0001
	Y	224 (8.4 %)	17 (1.6 %)	207 (13.0 %)	
Med combination at any visit	BB & ACE-I	662 (24.7 %)	181 (16.7 %)	481 (30.1 %)	<0.0001
	BB & ACE-I & MRA	148 (5.5 %)	26 (2.4 %)	122 (7.6 %)	<0.0001
	BB & ACE-I & Empagliflozin	11 (0.4 %)	2 (0.2 %)	9 (0.6 %)	0.2171
	BB & ACE-I & MRA & Empagliflozin	3 (0.1 %)	0 (0 %)	3 (0.2 %)	0.2775
	BB & ARB	289 (10.8 %)	114 (10.5 %)	175 (11.0 %)	0.7799
	BB & ARB & MRA	81 (3.0 %)	27 (2.5 %)	54 (3.4 %)	0.231
	BB & ARB & Empagliflozin	8 (0.3 %)	2 (0.2 %)	6 (0.4 %)	0.4864
	BB & ARB & MRA & Empagliflozin	2 (0.1 %)	2 (0.2 %)	0 (0 %)	0.163
	BB & Entresto	104 (3.9 %)	7 (0.6 %)	97 (6.1 %)	<0.0001
	BB & Entresto & MRA	59 (2.2 %)	6 (0.6 %)	53 (3.3 %)	<0.0001
	BB & Entresto & Empagliflozin	10 (0.4 %)	1 (0.1 %)	9 (0.6 %)	0.0568
	BB & Entresto & MRA & Empagliflozin	10 (0.4 %)	0 (0 %)	10 (0.6 %)	0.0074
	BB	829 (31.0 %)	393 (36.4 %)	436 (27.3 %)	<0.0001
	other combinations	491 (18.3 %)	231 (21.4 %)	260 (16.3 %)	0.001
	none of the above med	388 (14.5 %)	204 (18.9 %)	184 (11.5 %)	<0.0001
Adverse Reactions (hypoglycemia and urinary tract infection) at any visit	N	2366 (88.4 %)	931 (86.1 %)	1435 (89.9 %)	0.0033
	Y	311 (11.6 %)	150 (13.9 %)	161 (10.1 %)	
Hypoglycemia at any visit	N	2588 (96.7 %)	1043 (96.5 %)	1545 (96.8 %)	0.7316
	Y	89 (3.3 %)	38 (3.5 %)	51 (3.2 %)	
Urinary tract infection at any visit	N	2442 (91.2 %)	964 (89.2 %)	1478 (92.6 %)	0.0026
	Y	235 (8.8 %)	117 (10.8 %)	118 (7.4 %)	
Protein in UA at any visit	Negative	505 (33.5 %)	260 (39.4 %)	245 (28.9 %)	<0.0001
	Positive	1003 (66.5 %)	400 (60.6 %)	603 (71.1 %)	
EGFR at baseline visit	20–30	204 (7.6 %)	96 (8.9 %)	108 (6.8 %)	0.5542
	30–40	317 (11.9 %)	132 (12.2 %)	185 (11.6 %)	
	40–50	367 (13.7 %)	154 (14.3 %)	213 (13.4 %)	
	50–60	1452 (54.4 %)	576 (53.4 %)	876 (55.0 %)	
	60–70	83 (3.1 %)	30 (2.8 %)	53 (3.3 %)	
	70–80	53 (2.0 %)	21 (1.9 %)	32 (2.0 %)	
	80–90	76 (2.8 %)	28 (2.6 %)	48 (3.0 %)	
	90–100	63 (2.4 %)	25 (2.3 %)	38 (2.4 %)	
	100–110	39 (1.5 %)	11 (1.0 %)	28 (1.8 %)	
	110–120	11 (0.4 %)	5 (0.5 %)	6 (0.4 %)	
	120–130	5 (0.2 %)	1 (0.1 %)	4 (0.3 %)	
	130–140	1 (0.04 %)	0 (0 %)	1 (0.1 %)	

### 3.2 Prescription patterns of SGLT2 inhibitors

**Fig. 1:**
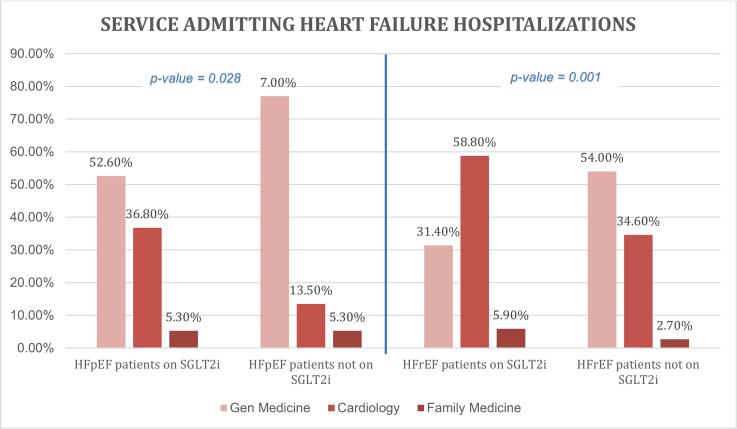
Depiction of services admitting heart failure hospitalizations.

Of the total 3548 encounters with 2677 unique numbers of patients, SGLT2i was prescribed in only 76 encounters (2.1 %): 21 encounters with 19 HFpEF patients and 55 encounters with 51 HFrEF patients respectively. Of the total number of patients with HFpEF that were prescribed an SGLT2i, 36.8 % were by a provider belonging to a cardiology service versus 57.9 % by a provider from a medicine service (either internal medicine or family medicine). However, of the total number of patients with HFrEF that were prescribed an SGLT2i, the cardiology service prescribed a majority (58.8 %), whereas the medicine services prescribed only 37.3 % at their baseline visit (Fig. 1).

Specifically, for HFpEF patients, those who were prescribed SGLT2i were more likely to be on an MRA (52.6 % vs. 13.7 %, p = 0.0001) and an ARNI (15.8 % vs. 1.3 %, p = 0.0027) compared to those who were not prescribed an SGLT2i. Moreover, similarly to HFpEF patients, HFrEF patients also showed that those prescribed an SGLT2i were more likely to be on an MRA (37.3 % vs. 21.3 %, p = 0.011) and ARNI (54.9 % vs. 11.6 %, p < 0.0001) compared to those who were not prescribed an SGLT2i. HFrEF patients who were prescribed an SGLT2i were also more likely to have T2DM (62.7 % vs. 45.1 %, p = 0.0191) and have a higher eGFR and BNP with a lower LVEF at baseline (60.51 vs. 54.41, p = 0.0472, 1507.04 vs. 1189.87, p = 0.035, 18.86 vs. 24.72, p = 0.003).

### 3.3 Adverse events

Regarding adverse effects, among HFrEF patients, those on an SGLT2i were less likely to have the presence of protein in their urine (53.3 % vs. 71.8 %, p = 0.0475) compared to those not on an SGLT2i. However, this finding was not statistically significant in HFpEF patients. In addition, other adverse effects related to the SGLT2i class such as hypoglycemia and presence of urinary tract infections were not significantly associated with SGLT2i use in both HFpEF and HFrEF groups.

## 4 Discussion

The benefits of SGLT2i use in patients with HFrEF has been well established in numerous studies [10], [11], [12]. Similar benefits have more recently been proven for patients with HFpEF as well. As seen in the EMPEROR-Preserved trial, a significant mortality benefit along with a reduced risk of re-hospitalization was observed, making the SGLT2i class of medications crucial for HFpEF patients in which treatment options remain limited [14]. While there has been an increase in prescription rates of SGLT2i over the past several years, the class still remains underutilized in daily clinical practice in both HFpEF and HFrEF populations [15]. According to our research, SGLT2i are especially underemployed in the HFpEF population as the prescription rate of SGLT2 inhibitors was significantly higher for the HFrEF population compared to the HFpEF population. Our findings also suggested that SGLT2i are seemingly prescribed as a final resort after the initiation of beta blocker, MRA, and ARNI treatment for patients with HFpEF. Although insurance regulations and cost have hindered its usage, an increase in provider awareness and utilization of SGLT2i may prove beneficial in overall outcomes [16].

Hesitation of providers to prescribe SGLT2i may be due to a lack of provider knowledge, hospital formulary restrictions as witnessed in our institution, and clinical inertia in which optimal medications are either never started or only slowly initiated in the outpatient setting [17], [18]. For example, our findings suggest that a major predictor of SGLT2i usage was being admitted to a cardiology service, which was similarly seen in other small-scale studies [19]. The fact that the SGLT2i prescription rate for HF is higher among cardiologists than that among primary care providers suggests a potential gap in awareness regarding this medication's efficacy in heart failure patients. However, it must be noted that our institutional policies prevent prescription of an SGLT2i without approval from a cardiologist, unless the patient is directly admitted to the cardiology service. This restriction makes it more difficult for general medicine services to prescribe an SGLT2i for HF patients and could help explain our findings. It is also worth mentioning that in our study, more SGLT2i prescriptions were made by the general medicine services (after obtaining approval from cardiology) than by the cardiology service for the HFpEF group. This finding could have been due to the fact that more patients with HFpEF were admitted to general medicine services than to a cardiology service.

Physician reluctance to provide disease-modifying agents in a patient group with poor adherence to medications and focus more on overall symptom relief likely also affects SGLT2i prescribing patterns [20]. Poor adherence to medications has been found to be more prevalent in the elderly, especially in patients with severe HF (NYHA Class IV), likely due to patient frailty [17], [21]. Our study exhibited this notion by showing that among the HFrEF group, patients with T2DM, lower baseline EF, and higher BNP were more likely to prescribed SGLT2i. Such findings were not present in HFpEF group, which could have been due to a smaller sample size in the HFpEF group. Our study also demonstrated that among the HFrEF group, those prescribed an SGLT2i had a higher baseline eGFR. Although this finding was not true for the HFpEF group, it may demonstrate prescribers' tendency to avoid SGLT2i in patients with low eGFR. It is worth discussing that the limitation of eGFR as a barrier to SGLT2i prescription in patients with HF and concomitant CKD was recently refuted. Dapagliflozin was the only SGLT2i approved for use in patients with an eGFR between 30 and 69 mL/min/1.73m^2^ as shown in the DAPA-CKD trial. Given the similar molecular structure, other SGLT2i medications such as empagliflozin would also prove efficacious in this cohort, and this was evidenced with the recent publication of the EMPA-KIDNEY trial that demonstrated lower risk of renal disease progression and death secondary to cardiovascular causes with empagliflozin use [22]. Moreover, the DELIVER trial demonstrated a benefit of dapagliflozin initiation regardless of recent hospitalization and showed no increase in adverse events in these patients [23]. Our study also demonstrated that usage of SGLT2i was not associated with adverse events including hypoglycemia and urinary tract infection in both HFpEF and HFrEF groups. It was found, however, that among patients who received SGLT2i therapy during hospitalization for acute decompensated heart failure (ADHF), a low eGFR was associated with a recurrent hospitalization for HF following discharge [23]. Early initiation of SGLT2i prior to the progression of CKD is therefore recommended for patients admitted for ADHF [24].

In addition to our findings, racial, gender, and socioeconomic disparities have also been associated with SGLT2i usage [21], [25], [26], [27]. These inequities are driven by decreased access to specialist care, provider bias and assumptions regarding medication adherence, and economic barriers to this drug class [21], [25]. Interestingly, it was found that low rates of SGLT2i prescription in black patients persisted even after adjustment of visits to cardiologists and endocrinologists, suggesting possibilities of inherent racism in clinical care [21]. Additionally, nationwide studies have demonstrated that the prescription rate of SGLT2i in females is lower compared to that in males [21], [26]. This pattern may be due to poorer provider communication with females, slower adoption of guideline therapies by females, and a higher risk of genital infections associated with this group [26], [27]. Income level is by far one of the greatest predictors of initiation and adherence to SGLT2i [4]. One study showed that owning commercial insurance versus Medicare was one factors strongly associated with SGLT2i prescription, which proves that medication cost is associated with different rates of use [4]. A commonly overlooked aspect of prescribing patterns may include provider bias on the ability of patients of different socioeconomic backgrounds to afford and adhere to this medication class.

Furthermore, financial and administrative burdens such as step therapy requirements of commercial plans and prior authorizations required by Medicaid plans also contribute to decreased prescription rates [4]. Although Medicare plans were found to be the most likely to cover at least one SGLT2i without either prior authorization or step therapy, one study demonstrated that the total estimated out-of-pocket costs for quadruple therapy in HFrEF (ARNI, beta blocker, MRA, and SGLT2i) would exceed $2000 annually under most Medicare plans [4]. Nevertheless, the SGLT2i class of medications has been proven to reduce costs to healthcare systems by reduced rates of hospitalization for heart failure [28]. Although insurance companies may not be incentivized due to high initial costs, studies have shown that these benefits are recognized in the long-term rather than short-term, underlining the need to increase the rate of its usage [28].

Methods to integrate prescription of SGLT2i by physicians may include a clinical practice algorithm that considers each HF patient's unique risk factors and existing medications to help guide the selection of SGLT2i therapy [16], [29]. The involvement of a well-coordinated team of healthcare providers, including cardiologists and primary care providers, will likely result in improved patient outcomes and increased satisfaction with care [1]. Additionally, early identification of patients at risk of HF would ensure targeting of SGLT2i to those who may achieve maximal benefits, irrespective of whether the patient needs additional glycemic control. Educational interventions through dissemination of guidelines via communication campaigns or structured didactic sessions has been associated with a proven improvement in prescription rates [30]. Electronic health record-based internal reminder systems can increase clinical ordering as evidenced in other large-scale studies [31]. However, this must take into consideration the effects of alert fatigue and patient follow-through and adherence. Finally, physician audit and feedback has been evidenced to lead to improvement in clinical practice, especially in physician prescribing, and could be applied to prescription of SGLT2i [32].

## 5 Limitations

Our study has several limitations. First, the nature of a retrospective study inherently leads to a lower quality of evidence when paralleled with a prospective study. Our study's findings represent the unique prescribing patterns of two small institutions within Florida but cannot be generalized to larger-scale hospitals because it of its retrospective design. Additionally, given that the EMPEROR trials were published relatively recently, our sample size was quite limited. As the results of these landmark trials become even more popularized with time, a larger study sample can be utilized, and similar studies can be conducted yearly moving forward.

## 6 Conclusion

Despite proven benefits of this class of medications as witnessed in large-scale clinical trials, the implementation of this drug class continues to be low. Efforts to increase SGLT2i prescription rate among providers may results in lower hospital readmission rates.

## Ethical statement

Hereby, I (Roshni Prakash, M.D.) consciously assure that for the manuscript “Prescribing Patterns of SGLT-2 Inhibitors for Patients with Heart Failure: A Two-Center Analysis” the following is fulfilled:

1) This material is the authors' own original work, which has not been previously published elsewhere.

2) The paper is not currently being considered for publication elsewhere.

3) The paper reflects the authors' own research and analysis in a truthful and complete manner.

4) The paper properly credits the meaningful contributions of co-authors and co-researchers.

5) The results are appropriately placed in the context of prior and existing research.

6) All sources used are properly disclosed (correct citation). Literally copying of text must be indicated as such by using quotation marks and giving proper reference.

7) All authors have been personally and actively involved in substantial work leading to the paper, and will take public responsibility for its content.

The violation of the Ethical Statement rules may result in severe consequences.

To verify originality, your article may be checked by the originality detection software iThenticate.

I agree with the above statements and declare that this submission follows the policies of Solid State Ionics as outlined in the Guide for Authors and in the Ethical Statement.

## Funding

None.

## Declaration of competing interest

The authors declare that they have no known competing financial interests or personal relationships that could have appeared to influence the work reported in this paper.

## Appendix A Supplementary data

Supplementary tables displaying patient characteristics. Image 1
