# Supplementary material for: Prescribing patterns of SGLT-2 inhibitors for patients with heart failure: A two-center analysis
Source: Am Heart J Plus. 2023 Mar 7;28:100286. doi: 10.1016/j.ahjo.2023.100286 (PMC10945956; doi:10.1016/j.ahjo.2023.100286)
Supplement: Supplementary file 1 — Supplementary tables displaying patient characteristics. [file mmc1.docx]

**Supplemental Tables**

Table 2. Characteristics of Patients on an SGLT2i with HFrEF and HFpEF

| Variable | Level | All | SGLT2 | | P-value |
| --- | --- | --- | --- | --- | --- |
|  |  |  | N | Y |  |
| Number of patients |  | 2677 | 2607 | 70 |  |
| Average number of encounters |  | 1.33±0.89 | 1.32±0.89 | 1.54±0.88 | 0.0395 |
| Length of stay (days) at baseline visit |  | 8.25±9.18 | 8.25±9.22 | 8.01±7.83 | 0.8037 |
| BNP at baseline visit |  | 973.28±1032.11 | 958.45±1018.42 | 1270.85±1248.60 | 0.0512 |
| Hemoglobin A1c at baseline visit |  | 6.62±1.81 | 6.61±1.81 | 7.08±1.91 | 0.2233 |
| EGFR at baseline visit |  | 53.91±16.53 | 53.79±16.43 | 58.28±19.62 | 0.0642 |
| LVEF at baseline visit |  | 38.82±20.26 | 39.23±20.13 | 30.55±21.24 | 0.0056 |
| HF type | HFrEF | 1596 (59.6%) | 1545 (59.3%) | 51 (72.9%) | 0.0305 |
|  | HFpEF | 1081 (40.4%) | 1062 (40.7%) | 19 (27.1%) |  |
| Admit Svc Division at baseline visit | General Medicine | 1678 (62.7%) | 1652 (63.4%) | 26 (37.1%) | <0.0001 |
|  | Cardiology | 715 (26.7%) | 678 (26.0%) | 37 (52.9%) |  |
|  | Family Medicine | 80 (3.0%) | 76 (2.9%) | 4 (5.7%) |  |
|  | Other | 204 (7.6%) | 201 (7.7%) | 3 (4.3%) |  |
| 180-Day Readmission | N | 2202 (82.3%) | 2154 (82.6%) | 48 (68.6%) | 0.004 |
|  | Y | 475 (17.7%) | 453 (17.4%) | 22 (31.4%) |  |
| SSDI Death | N | 2641 (98.7%) | 2571 (98.6%) | 70 (100.0%) |  |
|  | Y | 36 (1.3%) | 36 ( 1.4%) | 0 (0%) |  |
| LVEF at baseline visit | 10- | 33 (3.0%) | 27 (2.6%) | 6 (11.5%) | 0.009 |
|  | 10-20 | 283 (25.5%) | 266 (25.1%) | 17 (32.7%) |  |
|  | 20-30 | 157 (14.1%) | 147 (13.9%) | 10 (19.2%) |  |
|  | 30-40 | 128 (11.5%) | 125 (11.8%) | 3 (5.8%) |  |
|  | 40-45 | 48 (4.3%) | 48 (4.5%) | 0 (0%) |  |
|  | 45-50 | 56 (5.0%) | 55 (5.2%) | 1 (1.9%) |  |
|  | 50-60 | 266 (24.0%) | 256 (24.2%) | 10 (19.2%) |  |
|  | 60+ | 139 (12.5%) | 134 (12.7%) | 5 (9.6%) |  |
| Diabetes | N | 1408 (52.6%) | 1382 (53.0%) | 26 (37.1%) | 0.0123 |
|  | Y | 1269 (47.4%) | 1225 (47.0%) | 44 (62.9%) |  |
| SGLT2 Inhibitors at any visit | N | 2607 (97.4%) | 2607 (100.0%) | 0 (0%) | <0.0001 |
|  | Y | 70 (2.6%) | 0 (0%) | 70 (100.0%) |  |
| Empagliflozin (Jardiance) at any visit | N | 2609 (97.5%) | 2607 (100.0%) | 2 ( 2.9%) | <0.0001 |
|  | Y | 68 (2.5%) | 0 (0%) | 68 (97.1%) |  |
| Dapagliflozin (Farxiga) at any visit | N | 2674 (99.9%) | 2607 (100.0%) | 67 (95.7%) | <0.0001 |
|  | Y | 3 (0.1%) | 0 (0%) | 3 ( 4.3%) |  |
| Jardiance Dose (mg) | 10 | 65 (95.6%) | 0 (0%) | 65 (95.6%) |  |
|  | 25 | 3 (4.4%) | 0 (0%) | 3 (4.4%) |  |
| Alternative Medications (MRA, ACE_I, ARB, and Entresto) | N | 308 (11.5%) | 307 (11.8%) | 1 (1.4%) | 0.0129 |
|  | Y | 2369 (88.5%) | 2300 (88.2%) | 69 (98.6%) |  |
| Beta blocker | N | 532 (19.9%) | 527 (20.2%) | 5 (7.1%) | 0.0107 |
|  | Y | 2145 (80.1%) | 2080 (79.8%) | 65 (92.9%) |  |
| MRA | N | 2173 (81.2%) | 2132 (81.8%) | 41 (58.6%) | <0.0001 |
|  | Y | 504 (18.8%) | 475 (18.2%) | 29 (41.4%) |  |
| ACE_I | N | 1691 (63.2%) | 1644 (63.1%) | 47 (67.1%) | 0.5665 |
|  | Y | 986 (36.8%) | 963 (36.9%) | 23 (32.9%) |  |
| ARB | N | 2181 (81.5%) | 2126 (81.5%) | 55 (78.6%) | 0.6333 |
|  | Y | 496 (18.5%) | 481 (18.5%) | 15 (21.4%) |  |
| Sacubitril-Valsartan or Entresto | N | 2453 (91.6%) | 2414 (92.6%) | 39 (55.7%) | <0.0001 |
|  | Y | 224 (8.4%) | 193 (7.4%) | 31 (44.3%) |  |
| Med combination at any visit | BB & ACE_I | 662 (24.7%) | 660 (25.3%) | 2 (2.9%) | <0.0001 |
|  | BB & ACE_I & MRA | 148 (5.5%) | 146 (5.6%) | 2 (2.9%) | 0.4332 |
|  | BB & ACE_I & Empagliflozin | 11 (0.4%) | 0 (0%) | 11 (15.7%) | <0.0001 |
|  | BB & ACE_I & MRA & Empagliflozin | 3 (0.1%) | 0 (0%) | 3 ( 4.3%) | <0.0001 |
|  | BB & ARB | 289 (10.8%) | 287 (11.0%) | 2 (2.9%) | 0.0484 |
|  | BB & ARB & MRA | 81 (3.0%) | 80 (3.1%) | 1 (1.4%) | 0.7229 |
|  | BB & ARB & Empagliflozin | 8 (0.3%) | 0 (0%) | 8 (11.4%) | <0.0001 |
|  | BB & ARB & MRA & Empagliflozin | 2 (0.1%) | 0 (0%) | 2 ( 2.9%) | 0.0007 |
|  | BB & Entresto | 104 (3.9%) | 100 (3.8%) | 4 (5.7%) | 0.3481 |
|  | BB & Entresto & MRA | 59 (2.2%) | 54 (2.1%) | 5 (7.1%) | 0.0177 |
|  | BB & Entresto & Empagliflozin | 10 (0.4%) | 0 (0%) | 10 (14.3%) | <0.0001 |
|  | BB & Entresto & MRA & Empagliflozin | 10 (0.4%) | 0 (0%) | 10 (14.3%) | <0.0001 |
|  | BB | 829 (31.0%) | 823 (31.6%) | 6 (8.6%) | 0.0001 |
|  | other combinations | 491 (18.3%) | 462 (17.7%) | 29 (41.4%) | <0.0001 |
|  | none of the above med | 388 (14.5%) | 387 (14.8%) | 1 (1.4%) | 0.0029 |
| Adverse Reactions (hypoglycemia and urinary tract infection) at any visit | N | 2366 (88.4%) | 2300 (88.2%) | 66 (94.3%) | 0.1698 |
|  | Y | 311 (11.6%) | 307 (11.8%) | 4 (5.7%) |  |
| Hypoglycemia at any visit | N | 2588 (96.7%) | 2519 (96.6%) | 69 (98.6%) | 0.7293 |
|  | Y | 89 (3.3%) | 88 (3.4%) | 1 (1.4%) |  |
| Urinary tract infection at any visit | N | 2442 (91.2%) | 2376 (91.1%) | 66 (94.3%) | 0.4814 |
|  | Y | 235 (8.8%) | 231 (8.9%) | 4 (5.7%) |  |
| Protein in UA at any visit | Negative | 505 (33.5%) | 488 (33.2%) | 17 (43.6%) | 0.237 |
|  | Positive | 1003 (66.5%) | 981 (66.8%) | 22 (56.4%) |  |
| EGFR at baseline visit | 20 - 30 | 204 (7.6%) | 202 (7.8%) | 2 (2.9%) | 0.0245 |
|  | 30 - 40 | 317 (11.9%) | 307 (11.8%) | 10 (14.5%) |  |
|  | 40 - 50 | 367 (13.7%) | 354 (13.6%) | 13 (18.8%) |  |
|  | 50 - 60 | 1452 (54.4%) | 1430 (55.0%) | 22 (31.9%) |  |
|  | 60 - 70 | 83 (3.1%) | 76 (2.9%) | 7 (10.1%) |  |
|  | 70 - 80 | 53 (2.0%) | 50 (1.9%) | 3 (4.3%) |  |
|  | 80 - 90 | 76 (2.8%) | 69 (2.7%) | 7 (10.1%) |  |
|  | 90 - 100 | 63 (2.4%) | 61 (2.3%) | 2 (2.9%) |  |
|  | 100 - 110 | 39 (1.5%) | 36 (1.4%) | 3 (4.3%) |  |
|  | 110 - 120 | 11 (0.4%) | 11 (0.4%) | 0 (0%) |  |
|  | 120 - 130 | 5 (0.2%) | 5 (0.2%) | 0 (0%) |  |
|  | 130 - 140 | 1 (0.04%) | 1 (0.04%) | 0 (0%) |  |

Table 3. Characteristics of Patients with HFpEF and an SGLT2i

| Variable | Level | All | SGLT2 | | P-value |
| --- | --- | --- | --- | --- | --- |
|  |  |  | N | Y |  |
| Number of patients |  | 1081 | 1062 | 19 |  |
| Average number of encounters |  | 1.25±0.71 | 1.24±0.71 | 1.47±0.70 | 0.0128 |
| Length of stay (days) at baseline visit |  | 8.07±8.05 | 8.08±8.09 | 7.47±5.41 | 0.328 |
| BNP at baseline visit |  | 611.02±672.37 | 609.53±662.50 | 654.11±937.51 | 0.7542 |
| Hemoglobin A1c at baseline visit |  | 6.52±1.64 | 6.51±1.64 | 6.78±2.10 | 0.8542 |
| EGFR at baseline visit |  | 52.89±16.31 | 52.90±16.31 | 51.94±16.66 | 0.9337 |
| LVEF at baseline visit |  | 59.83±7.58 | 59.85±7.62 | 59.39±6.33 | 0.9293 |
| Admit Svc Division at baseline visit | General Medicine | 828 (76.6%) | 818 (77.0%) | 10 (52.6%) | 0.028 |
|  | Cardiology | 150 (13.9%) | 143 (13.5%) | 7 (36.8%) |  |
|  | Family Medicine | 35 (3.2%) | 34 (3.2%) | 1 (5.3%) |  |
|  | Other | 68 (6.3%) | 67 (6.3%) | 1 (5.3%) |  |
| 180-Day Readmission | N | 924 (85.5%) | 911 (85.8%) | 13 (68.4%) | 0.0454 |
|  | Y | 157 (14.5%) | 151 (14.2%) | 6 (31.6%) |  |
| SSDI Death | N | 1072 (99.2%) | 1053 (99.2%) | 19 (100.0%) | 1 |
|  | Y | 9 (0.8%) | 9 ( 0.8%) | 0 (0%) |  |
| LVEF at baseline visit |  |  |  |  | 0.9336 |
|  | 45-50 | 55 (12.2%) | 54 (12.4%) | 1 (6.7%) |  |
|  | 50-60 | 259 (57.3%) | 250 (57.2%) | 9 (60.0%) |  |
|  | 60+ | 138 (30.5%) | 133 (30.4%) | 5 (33.3%) |  |
| Diabetes | N | 541 (50.0%) | 534 (50.3%) | 7 (36.8%) | 0.3524 |
|  | Y | 540 (50.0%) | 528 (49.7%) | 12 (63.2%) |  |
| SGLT2 Inhibitors at any visit | N | 1062 (98.2%) | 1062(100.0%) | 0 (0%) | <0.0001 |
|  | Y | 19 (1.8%) | 0 (0%) | 19 (100.0%) |  |
| Empagliflozin (Jardiance) at any visit | N | 1062 (98.2%) | 1062 (100.0%) | 0 (0%) | <0.0001 |
|  | Y |  |  |  |  |
| Alternative Medications (MRA, ACE_I, ARB, and Entresto) | N | 176 (16.3%) | 175 (16.5%) | 1 (5.3%) | 0.3419 |
|  | Y | 905 (83.7%) | 887 (83.5%) | 18 (94.7%) |  |
| Beta blocker | N | 319 (29.5%) | 317 (29.8%) | 2 (10.5%) | 0.1149 |
|  | Y | 762 (70.5%) | 745 (70.2%) | 17 (89.5%) |  |
| MRA | N | 925 (85.6%) | 916 (86.3%) | 9 (47.4%) | 0.0001 |
|  | Y | 156 (14.4%) | 146 (13.7%) | 10 (52.6%) |  |
| ACE_I | N | 782 (72.3%) | 768 (72.3%) | 14 (73.7%) | 1 |
|  | Y | 299 (27.7%) | 294 (27.7%) | 5 (26.3%) |  |
| ARB | N | 877 (81.1%) | 864 (81.4%) | 13 (68.4%) | 0.1486 |
|  | Y | 204 (18.9%) | 198 (18.6%) | 6 (31.6%) |  |
| Sacubitril-Valsartan or Entresto | N | 1064 (98.4%) | 1048 (98.7%) | 16 (84.2%) | 0.0027 |
|  | Y | 17 (1.6%) | 14 (1.3%) | 3 (15.8%) |  |
| Adverse Reactions (hypoglycemia and urinary tract infection) at any visit | N | 931 (86.1%) | 913 (86.0%) | 18 (94.7%) | 0.4994 |
|  | Y | 150 (13.9%) | 149 (14.0%) | 1 (5.3%) |  |
| Hypoglycemia at any visit | N | 1043 (96.5%) | 1024 (96.4%) | 19 (100.0%) | 1 |
|  | Y | 38 (3.5%) | 38 ( 3.6%) | 0 (0%) |  |
| Urinary tract infection at any visit | N | 964 (89.2%) | 946 (89.1%) | 18 (94.7%) | 0.7118 |
|  | Y | 117 (10.8%) | 116 (10.9%) | 1 (5.3%) |  |
| Protein in UA at any visit | Negative | 260 (39.4%) | 257 (39.5%) | 3 (33.3%) | 1 |
|  | Positive | 400 (60.6%) | 394 (60.5%) | 6 (66.7%) |  |
| EGFR at baseline visit | 20 - 30 | 96 (8.9%) | 95 (9.0%) | 1 (5.6%) | 0.0245 |
|  | 30 - 40 | 132 (12.2%) | 130 (12.3%) | 2 (11.1%) |  |
|  | 40 - 50 | 154 (14.3%) | 147 (13.9%) | 7 (38.9%) |  |
|  | 50 - 60 | 576 (53.4%) | 573 (54.0%) | 3 (16.7%) |  |
|  | 60 - 70 | 30 (2.8%) | 27 (2.5%) | 3 (16.7%) |  |
|  | 70 - 80 | 21 (1.9%) | 21 (2.0%) | 0 (0%) |  |
|  | 80 - 90 | 28 (2.6%) | 26 (2.5%) | 2 (11.1%) |  |
|  | 90 - 100 | 25 (2.3%) | 25 (2.4%) | 0 (0%) |  |
|  | 100 - 110 | 11 (1.0%) | 11 (1.0%) | 0 (0%) |  |
|  | 110 - 120 | 5 (0.5%) | 5 (0.5%) | 0 (0%) |  |
|  | 120 - 130 | 1 (0.1%) | 1 (0.1%) | 0 (0%) |  |
|  | 130 - 140 | 0 (0%) | 0 (0%) | 0 (0%) |  |

Table 4. Characteristics of Patients with HFrEF and an SGLT2i

| Variable | Level | All | SGLT2 | | P-value |
| --- | --- | --- | --- | --- | --- |
|  |  |  | N | Y |  |
| Number of patients |  | 1596 | 1545 | 51 |  |
| Average number of encounters |  | 1.38±0.99 | 1.37±0.99 | 1.57±0.94 | 0.0199 |
| Length of stay (days) at baseline visit |  | 8.37±9.87 | 8.37±9.92 | 8.22±8.60 | 0.7828 |
| BNP at baseline visit |  | 1207.81±1150.27 | 1189.87±1140.44 | 1507.04±1279.99 | 0.035 |
| Hemoglobin A1c at baseline visit |  | 6.68±1.90 | 6.67±1.90 | 7.15±1.91 | 0.162 |
| EGFR at baseline visit |  | 54.60±16.66 | 54.41±16.50 | 60.51±20.23 | 0.0472 |
| LVEF at baseline visit |  | 24.39±11.88 | 24.72±11.82 | 18.86±11.68 | 0.003 |
| Admit Svc Division at baseline visit | General Medicine | 850 (53.3%) | 834 (54.0%) | 16 (31.4%) | 0.001 |
|  | Cardiology | 565 (35.4%) | 535 (34.6%) | 30 (58.8%) |  |
|  | Family Medicine | 45 (2.8%) | 42 (2.7%) | 3 (5.9%) |  |
|  | Other | 136 (8.5%) | 134 (8.7%) | 2 (3.9%) |  |
| 180-Day Readmission | N | 1278 (80.1%) | 1243 (80.5%) | 35 (68.6%) | 0.0572 |
|  | Y | 318 (19.9%) | 302 (19.5%) | 16 (31.4%) |  |
| SSDI Death | N | 1569 (98.3%) | 1518 (98.3%) | 51 (100.0%) | 1 |
|  | Y | 27 (1.7%) | 27 ( 1.7%) | 0 (0%) |  |
| LVEF at baseline visit | 10- | 33 (5.0%) | 27 (4.3%) | 6 (16.2%) | 0.0156 |
|  | 10-20 | 283 (43.0%) | 266 (42.8%) | 17 (45.9%) |  |
|  | 20-30 | 157 (23.9%) | 147 (23.7%) | 10 (27.0%) |  |
|  | 30-40 | 128 (19.5%) | 125 (20.1%) | 3 (8.1%) |  |
|  | 40-45 | 48 (7.3%) | 48 (7.7%) | 0 (0%) |  |
| Diabetes | N | 867 (54.3%) | 848 (54.9%) | 19 (37.3%) | 0.0191 |
|  | Y | 729 (45.7%) | 697 (45.1%) | 32 (62.7%) |  |
| SGLT2 Inhibitors at any visit | N | 1545 (96.8%) | 1545 (100.0%) | 0 (0%) | <0.0001 |
|  | Y | 51 (3.2%) | 0 (0%) | 51 (100.0%) |  |
| Empagliflozin (Jardiance) at any visit | N | 1547 (96.9%) | 1545 (100.0%) | 2 ( 3.9%) | <0.0001 |
|  | Y | 49 (3.1%) | 0 (0%) | 49 (96.1%) |  |
| Alternative Medications (MRA, ACE_I, ARB, and Entresto) | N | 132 (8.3%) | 132 ( 8.5%) | 0 (0%) | 0.0186 |
|  | Y | 1464 (91.7%) | 1413 (91.5%) | 51 (100.0%) |  |
| Beta blocker | N | 213 (13.3%) | 210 (13.6%) | 3 (5.9%) | 0.1664 |
|  | Y | 1383 (86.7%) | 1335 (86.4%) | 48 (94.1%) |  |
| MRA | N | 1248 (78.2%) | 1216 (78.7%) | 32 (62.7%) | 0.011 |
|  | Y | 348 (21.8%) | 329 (21.3%) | 19 (37.3%) |  |
| ACE_I | N | 909 (57.0%) | 876 (56.7%) | 33 (64.7%) | 0.3209 |
|  | Y | 687 (43.0%) | 669 (43.3%) | 18 (35.3%) |  |
| ARB | N | 1304 (81.7%) | 1262 (81.7%) | 42 (82.4%) | 1 |
|  | Y | 292 (18.3%) | 283 (18.3%) | 9 (17.6%) |  |
| Sacubitril-Valsartan or Entresto | N | 1389 (87.0%) | 1366 (88.4%) | 23 (45.1%) | <0.0001 |
|  | Y | 207 (13.0%) | 179 (11.6%) | 28 (54.9%) |  |
| Adverse Reactions (hypoglycemia and urinary tract infection) at any visit | N | 1435 (89.9%) | 1387 (89.8%) | 48 (94.1%) | 0.437 |
|  | Y | 161 (10.1%) | 158 (10.2%) | 3 (5.9%) |  |
| Hypoglycemia at any visit | N | 1545 (96.8%) | 1495 (96.8%) | 50 (98.0%) | 1 |
|  | Y | 51 (3.2%) | 50 (3.2%) | 1 (2.0%) |  |
| Urinary tract infection at any visit | N | 1478 (92.6%) | 1430 (92.6%) | 48 (94.1%) | 1 |
|  | Y | 118 (7.4%) | 115 (7.4%) | 3 (5.9%) |  |
| Protein in UA at any visit | Negative | 245 (28.9%) | 231 (28.2%) | 14 (46.7%) | 0.0475 |
|  | Positive | 603 (71.1%) | 587 (71.8%) | 16 (53.3%) |  |
| EGFR at baseline visit | 20 - 30 | 108 (6.8%) | 107 (6.9%) | 1 (2.0%) | 0.046 |
|  | 30 - 40 | 185 (11.6%) | 177 (11.5%) | 8 (15.7%) |  |
|  | 40 - 50 | 213 (13.4%) | 207 (13.4%) | 6 (11.8%) |  |
|  | 50 - 60 | 876 (55.0%) | 857 (55.6%) | 19 (37.3%) |  |
|  | 60 - 70 | 53 (3.3%) | 49 (3.2%) | 4 (7.8%) |  |
|  | 70 - 80 | 32 (2.0%) | 29 (1.9%) | 3 (5.9%) |  |
|  | 80 - 90 | 48 (3.0%) | 43 (2.8%) | 5 (9.8%) |  |
|  | 90 - 100 | 38 (2.4%) | 36 (2.3%) | 2 (3.9%) |  |
|  | 100 - 110 | 28 (1.8%) | 25 (1.6%) | 3 (5.9%) |  |
|  | 110 - 120 | 6 (0.4%) | 6 (0.4%) | 0 (0%) |  |
|  | 120 - 130 | 4 (0.3%) | 4 (0.3%) | 0 (0%) |  |
|  | 130 - 140 | 1 (0.1%) | 1 (0.1%) | 0 (0%) |  |
